# Accessibility to health care facilities in Montreal Island: an application of relative accessibility indicators from the perspective of senior and non-senior residents

**DOI:** 10.1186/1476-072X-9-52

**Published:** 2010-10-25

**Authors:** Antonio Paez, Ruben G Mercado, Steven Farber, Catherine Morency, Matthew Roorda

**Affiliations:** 1 School of Geography and Earth Sciences, McMaster University, Hamilton Ontario, Canada; 2 Cities Centre, University of Toronto, Toronto, Ontario, Canada; 3 Department of Geography, Ryerson University, Toronto, Ontario, Canada; 4 Département des génies civil, géologique et des mines, École Polytechnique, Montréal, Québec, Canada; 5 Department of Civil Engineering, University of Toronto, Toronto, Canada

## Abstract

**Background:**

Geographical access to health care facilities is known to influence health services usage. As societies age, accessibility to health care becomes an increasingly acute public health concern. It is known that seniors tend to have lower mobility levels, and it is possible that this may negatively affect their ability to reach facilities and services. Therefore, it becomes important to examine the mobility situation of seniors vis-a-vis the spatial distribution of health care facilities, to identify areas where accessibility is low and interventions may be required.

**Methods:**

Accessibility is implemented using a cumulative opportunities measure. Instead of assuming a fixed bandwidth (i.e. a distance threshold) for measuring accessibility, in this paper the bandwidth is defined using model-based estimates of average trip length. Average trip length is an all-purpose indicator of individual mobility and geographical reach. Adoption of a spatial modelling approach allows us to tailor these estimates of travel behaviour to specific locations and person profiles. Replacing a fixed bandwidth with these estimates permits us to calculate customized location- and person-based accessibility measures that allow inter-personal as well as geographical comparisons.

**Data:**

The case study is Montreal Island. Geo-coded travel behaviour data, specifically average trip length, and relevant traveller's attributes are obtained from the Montreal Household Travel Survey. These data are complemented with information from the Census. Health care facilities, also geo-coded, are extracted from a comprehensive business point database. Health care facilities are selected based on Standard Industrial Classification codes 8011-21 (Medical Doctors and Dentists).

**Results:**

Model-based estimates of average trip length show that travel behaviour varies widely across space. With the exception of seniors in the downtown area, older residents of Montreal Island tend to be significantly less mobile than people of other age cohorts. The combination of average trip length estimates with the spatial distribution of health care facilities indicates that despite being more mobile, suburban residents tend to have lower levels of accessibility compared to central city residents. The effect is more marked for seniors. Furthermore, the results indicate that accessibility calculated using a fixed bandwidth would produce patterns of exposure to health care facilities that would be difficult to achieve for suburban seniors given actual mobility patterns.

**Conclusions:**

The analysis shows large disparities in accessibility between seniors and non-seniors, between urban and suburban seniors, and between vehicle owning and non-owning seniors. This research was concerned with potential accessibility levels. Follow up research could consider the results reported here to select case studies of actual access and usage of health care facilities, and related health outcomes.

## Background and Objectives

Access to health care is a multi-dimensional concept that involves financial accessibility, availability, acceptability, and geographical accessibility [1]. Studies in the US have shown that usage of health care services is affected by the ownership of health insurance (employer or public) as well as by the out-of-pocket cost of care obligated under various types of insurance [2-6]. Insurance coverage has been found to increase survival chances and significantly reduce the odds of transitions from independence to disability [7]. Contrariwise, lack of coverage is associated with negative outcomes, including declines in function, the emergence of otherwise preventable health issues, and even premature mortality [8]. Coverage, or more accurately lack thereof, is a significant problem in the US mostly for poor or near poor people, and for many in poor or developing countries [1,8]. Most countries in the developed world, including Canada, consider provision of health care a citizen right and accordingly strive to provide universal coverage. In the US, universal coverage has long been afforded only to certain population segments, such as seniors covered by Medicare.

In such cases where financial accessibility issues are obviated by insurance, other factors could better explain issues related to health care utilization. There is at present a growing body of evidence which shows that besides financial responsibilities, barriers to utilization are related to the socio-demographic characteristics of the individual and the environment within which the individual uses health services. Included in the individual-based barriers are those that concern a person's age, race, income, gender, education and subjective satisfaction with service providers [9-14]. Lack of access to a vehicle, a factor closely related to income, age, and gender, has been found to restrict access to health and social care resources [15]. There have also been studies that look at the relational aspects of the individual and found some connections with the size and closeness of their social networks [16-18]. Barriers to receiving health care also include cultural and linguistic factors [12]. A recent study among older Chinese immigrants in Canada confirms most of these factors showing the following significant barriers to health service: being female, single, shorter length of residency, income, social network, health beliefs, and their self-identification as Canadian [19]. Other important factors that interact with use of health care services include their quality, the perception of the provider, and the past experience with the service or re-treatment [20]. A review of evidence from studies in the US underline the increasing diversity and unmet demand of the older adult population for oral care, and emerging dental workforce issues including training opportunities in gerontology and geriatrics for dental practitioners [21].

With regards to the environment within which a person can avail him/herself of care, the location and distribution of health care services and the quality of transportation have also received increased attention. These environmental conditions have been variously termed in the literature as "spatial factors" [22] or "structural or physical barriers" from the patient's standpoint [12]. Accessibility, defined as the travel impedance between patient location and the locations where care is delivered, comes to the fore as an approach to understand the geographical dimension of health care [23]. Despite being of obvious interest, until recently relatively little was known about the geographical accessibility to health care. As foretold by Guagliardo [23], however, this situation was bound to change with continued advances in geospatial analysis, as well as increased availability and affordability of geographic information and software. Indeed, during the past few years, a number of studies have contributed to advance the methods used to measure geographical accessibility. This includes more refined approaches to match provision of services and population coverage [24-26], the creation of specialized software [27], the use of gravity models [28], investigations of measurement and error [29], and the introduction of optimization techniques [30].

In addition to methodological advances, progress has also been seen in terms of filling the knowledge gap regarding the situation and implications of geographical accessibility to healthcare. Several studies already provide evidence of the effect of distance to facility on service utilization. For instance, a study in the UK analyzed patient choice policy (i.e. people can choose the hospital where they would like to be treated), and found a negative relationship between the use of services and distance [20]. Research in Italy also found that radiotherapy utilization tends to decreases with increasing distance to the nearest facility [31].

Distance has been shown to matter in previous research. Alone as a barrier, however, distance does not fully explain accessibility, since transportation and mobility factors are also influential. In particular, while the individual and environmental factors that may pose barriers to health care have been independently studied, there has been only limited research into the way the individual and his/her environment may interact to influence accessibility levels. The relevance of these interactions becomes particularly poignant for the case of seniors, a population segment that for all its remarkable heterogeneity [32], typically tends to be less mobile [33-39]. Seniors are particularly vulnerable to mobility disruptions when driving reduction or cessation eventually occur [40,41]. Regardless of whether driving reduction or cessation results from self-censoring or medical conditions, the effect is to limit the range and frequency of activities outside the home, which may include visits to a doctor or a dentist. Public transport becomes an important alternative to the automobile [42], although transit is often an imperfect substitute in terms of matching the levels of mobility provided by the car [35].

The objective of this study is to investigate the status of accessibility to health care of senior and non-senior residents in Montreal Island.

The contributions of the paper are twofold. First, this research adds incrementally to the evidence base regarding accessibility to health care facilities in Canada, particularly from the perspective of seniors. Secondly, accessibility calculations frequently assume a fixed bandwidth (a distance or travel time threshold), and therefore provide measures that depend exclusively on the spatial distribution of facilities, but are insensitive to location and personal factors. Geographically, however, there is evidence that people use space differently depending on their situation and location. Travelling for 30 minutes may be a completely different, and considerably more burdensome, experience for someone who is 70 years old, compared to someone who is 20 years old. The experience for a 20 year old may also be completely different in the suburbs or the centre of a city. Therefore, the second contribution of this paper therefore is to demonstrate the use of relative accessibility deprivation indicators [43] for the analysis of accessibility to health care. Relative accessibility indicators are calculated using model-based estimates of personal average trip length. This is an all-purpose indicator of individual mobility and range, and provides a useful proxy for activity spaces, or the "spaces of daily life" [36]. Comparison with fixed bandwidth accessibility measures reveals, in fact, that assuming invariant personal and geographically-based behaviour can lead to estimates of accessibility to health care that are at times overly optimistic, and difficult to meet based on actual mobility patterns, or overly pessimistic, and therefore misleading in terms of actual needs.

## Methods

### Measuring Accessibility and Relative Accessibility Indicators

A number of papers exist that extensively review the concept of accessibility from a general transportation perspective [44-46] and from a health geography perspective as well [23,29]. A family of accessibility measures frequently discussed in these literatures is given by:

(1) $$A^{k}\left(i\right)=\sum_{j}W_{j}^{k}K\left(\frac{c_{ij}}{\gamma }\right)$$

In the equation above, accessibility *A *to opportunity of type *k *from the perspective of location *i*, is a function of the number of opportunities of the same type available at location *j*, discounted by the travel impedance (itself a function of cost *c_ij_*) of reaching that location. *K*(·) is a distance-decay function with a rate of decay controlled by bandwidth parameter *γ*. According to the equation, accessibility increases proportionally with the number of opportunities and decreases as the distance to these opportunities increases. Other things being equal, accessibility also decreases as the bandwidth parameter becomes smaller.

For this research we use the following cumulative opportunities measure, obtained when the distance-decay function is binary:

(2) $$K=\{\begin{array}{cc} 1 & \text{if }c_{ij}\leq \gamma \\ 0 & \text{otherwise} \end{array}$$

According to this formulation, all opportunities located within the threshold defined by *γ *are deemed to be accessible. The accessibility measure then becomes:

(3) $$A^{k}\left(i\right)=\sum_{j}W_{j}^{k}K\left(c_{ij}\leq \gamma \right)$$

where *K*(·) is an indicator function that takes the value of 1 if the logical statement in the argument of the function is true (i.e. if the cost of reaching *j *from *i *does not exceed the value of the bandwidth parameter) and 0 otherwise. The indicator in is attractive because it has an intuitive interpretation in terms of the number of opportunities that can be reached. Other distance-decay functions (e.g. inverse distance or negative exponential) produce smoother map patterns [47], but require the use of additional parameters and introduce distance- (or cost-) discounted schemes that are more difficult to interpret. Previous research has shown that cumulative opportunity measures tend to be highly correlated regardless of the distance-decay function used [45], and in the end we favour simplicity and interpretability in our selection of an accessibility indicator.

Calculation of accessibility measures of the family represented by equation generally requires selection of a bandwidth parameter. A number of different values are reported in the literature on accessibility to health care. Guagliardo et al. [48], for example, use a bandwidth of 4.8 km for the analysis of pediatric providers. This value is selected based on information provided by an earlier study of urban black people by Shannon et al. [49]. Apparicio et al. [29] in their study of health services in Montreal explore distance bandwidths of 500, 1000, and 2000 m. Travel time has also been used instead of distance. Luo and Wang [50], for instance, use a 30 min driving time threshold, following a suggestion by Lee [51] in earlier work that reviewed criteria used to designate shortage areas. The same 30 min driving threshold is used by Gu et al. [30] in their analysis of accessibility to cancer screening clinics in Alberta, and by Wang et al. [52] in their study of late-stage breast cancer and health care access. These latter two papers cite as a reason for selecting this bandwidth a standard used by the U.S. Department of Health and Human Services to define service areas. In terms of distance, thirty minutes driving time converts to approximately 27.5 km using a speed limit of 55 km/h in effect in many urban areas in Canada and around the world. Use of this standard can be questioned on at least two grounds. First, from an equity perspective, it essentially ignores all those without access to a vehicle - a serious inadequacy considering that many seniors eventually face driving limitations or cessation. And secondly, it may also overestimate the willingness to travel of even people with a vehicle: research by Haynes et al. [53]; [particularly Table 1] in England indicates that while some patients will bypass their nearest practice, relatively few of them will travel by car longer than 15 min to go to a more distant practice. If nothing else, this reveals a preference for more proximate health services.

As the preceding review suggests, there does not appear to be a consensus on appropriate bandwidth values. A point of agreement perhaps is that the bandwidth should be selected based on empirical information about mobility patterns, to account for variability in transport burden according to socio-economic status and neighbourhood characteristics [48]; [p. 281]. In essence, this argues for the use of flexible bandwidths in accessibility analysis to better reflect the individual circumstances of typical travellers. A proposal is to use flexible bandwidths *γ_pi _*specific to location (*i*) and personal profile (*p*) as follows [43]:

(4) $$A_{p}^{k}\left(i\right)=\sum_{j}W_{j}^{k}K\left(d_{ij}\leq \gamma_{pi}\right)$$

Introduction of flexible bandwidths means that accessibility levels can potentially vary between different individuals even at the same location (e.g. seniors may not experience their environment in the same way as younger people). Moreover, it is possible to account for situations where the burden of transportation is different even for identical individuals but at different locations. An important implication of using flexible bandwidths is that it becomes possible to conduct more refined analyses of accessibility that consider accessibility relationships, therefore the term relative accessibility deprivation indicators [43]. Use of a fixed bandwidth, given its lack of sensitivity to "ecological circumstances" [48]; [p. 281] precludes this type of relational analysis. Flexible bandwidths make it possible to define indicators such as the following measure of relative accessibility between two individuals, *p *and *q*, belonging to different population segments:

(5) $$R_{pq}^{k}\left(i\right)=\frac{A_{p}^{k}\left(i\right)}{A_{q}^{k}\left(i\right)}=\frac{\sum_{j}W_{j}^{k}K\left(d_{ij}^{k}\leq \gamma_{pi}\right)}{\sum_{j}W_{j}^{k}K\left(d_{ij}^{k}\leq \gamma_{qi}\right)}$$

The indicator above is a measure of how many more (or less) opportunities can an individual of type *p *at *i *reach, relative to the opportunities that an individual of type *q *at the same location can reach. The indicator is a proportion that takes a value of 1 when there is accessibility parity (both individuals have access to identical number of opportunities).

A mechanism for selecting flexible bandwidths is proposed [43] based on the use of empirically-based estimates of average trip length, after the analysis of distance travelled in a selection of Canadian cities [36]. Average trip length considering all purposes is a general indicator of overall mobility and a proxy for activity spaces (see Morency et al. [36], and before them Schönfelder and Axhausen [54]). This measure is likely an imperfect approximation of the distance that a person may be willing to travel for a specific purpose. Nevertheless, it is useful benchmark for accessibility measurements, in the sense that it captures opportunities available within the distance covered by a typical trip. Opportunities located at a longer distance would imply increasingly atypical trips with a higher cost than usual. The basic idea is to employ this indicator of mobility in conjunction with a geographical modelling approach to obtain a fine grained description of travel behaviour (time or distance travelled) to replace *γ_pi _*in Equation. A modelling approach, in addition to enabling relative accessibility analyses, also offers the advantage that estimates, say of average trip length by individual *p *and location *i *${\hat{d}}_{ip}$, are net of any confounding effects, for instance between aging, low income, and/or lack of a vehicle. This is discussed more fully below.

### The Expansion Method and Estimates of Average Trip Length

Estimates of average trip length can be obtained in a multivariate framework through the use of regression techniques. Average trip length *d*, defined as total distance travelled divided by number of trips, is a basic indicator of individual mobility. In a modelling framework, *d *is deemed to be a function of a set of explanatory factors, selected for their theoretical, practical, or policy relevance. Trip length is known to display a long-tailed distribution, and therefore a logarithmic transformation is typically used to compress the scale of the variable. The relevant model then becomes:

(6) $$\log\left(d_{i}\right)=\beta_{0}+\sum_{j}X_{ij}\beta_{j}+\varepsilon_{i}$$

This is a common log-linear model with regression coefficients *β *that can be estimated using conventional ordinary least squares under the usual assumptions for the residual terms *ε_i_*. A more general form of the model can be obtained following the principles of Casetti's expansion approach [55]. The expansion method belongs to a class of local spatial analysis techniques that include multi-level models and geographically weighted regression. The advantages of the expansion method in this specific type of applications are discussed in detail by Roorda et al. [38] and Morency et al. [36]. More concretely, the expansion method is used to derive models that incorporate variables of substantive interest as part of an initial model, as well as contextual factors as part of an expanded model. In geographical analysis, the contextual factors are usually the spatial coordinates of the observations, say *u_i _*and *v_i_*. An expanded model may incorporate interactions between the contextual factors (i.e. the coordinates) and all or some variables of substantive interest as follows:

(7) $$\log\left(d_{i}\right)=\sum_{j}X_{ij}\beta_{j}+\sum_{s}Z_{is}\theta_{is}+\varepsilon_{i}$$

Note that the expanded coefficients *θ *now are specific to location *i*. The constant terms would be spatially invariant if a vector of 1's is included as an *X*, or spatially varying if included as a *Z*. The expansion takes a linear form if we define:

(8) $$\theta_{is}=\theta_{s1}+\theta_{s2}u_{i}+\theta_{s3}v_{i}$$

A quadratic form is given by:

(9) $$\theta_{is}=\theta_{s1}1+\theta_{s2}u_{i}^{2}+\theta_{s3}u_{i}+\theta_{s4}u_{i}v_{i}+\theta_{s5}v_{i}+\theta_{s6}v_{i}^{2}$$

Higher order expansions are of course possible, but carry the risk of increased collinearity.

The operation of the expansion method is perhaps more easily understood if illustrated with an example. Consider the following initial model, where (log-transformed) average trip length is assumed to be a function of three variables, say income *I *(in $1000s), senior status *S *(= 1 if senior), and vehicle ownership *V *(= 1 if own):

(10) $$\log\left(d_{i}\right)=\beta_{0}+\beta_{1}I+\theta_{1}S+\theta_{2}V+\varepsilon_{i}$$

Furthermore, two variables (*S *and *V*) are of geographical interest and candidates for expansion. The initial model assumes that the relationships between average trip length and the explanatory variables are spatially invariant. For instance, the model assumes that, other things being equal, the travel behavior of seniors is the same whether in the suburbs or in the city centre. Use of a linear expansion for the coefficients *θ *in contrast leads to the following terminal model:

(11) $$\log\left(d_{i}\right)=\beta_{0}+\beta_{1}I_{i}+\theta_{11}S_{i}+\theta_{12}u_{i}S_{i}+\theta_{13}v_{i}S_{i}+\theta_{21}V_{i}+\theta_{22}u_{i}V_{i}+\theta_{23}v_{i}V_{i}+\varepsilon_{i}$$

which includes, in addition to spatially invariant effects for seniority and vehicle ownership (i.e. *θ*_11 _and *θ*_21 _respectively), geographical contextual effects. It is now possible to assess the effect on average trip distance of being a senior or owning a vehicle at different locations, since the net effect of these variables is a function of the spatial coordinates of the observation:

(12) $$\theta_{i1}S_{i}=\left(\theta_{11}+\theta_{12}u_{i}+\theta_{13}v_{i}\right)S_{i}$$

(13) $$\theta_{i2}V_{i}=\left(\theta_{21}+\theta_{22}u_{i}+\theta_{23}v_{i}\right)V_{i}$$

Models with expanded coefficients, being nothing other than interactions between substantive and contextual variables, can be estimated by means of ordinary least squares. The significance of the coefficients can be assessed using their *t*-scores or *p*-values, and the goodness-of-fit of the model summarized as the variance explained by means of the usual coefficient of determination *R*^2^.

After the coefficients of the model have been estimated, estimates of distance can be obtained for a specific location and personal profile by judicious manipulation of the inputs to the model. For instance, consider the following personal profile: (*Y*)oung (non-senior, therefore *S_i _*= 0), (*H*)igh Income (*I_i _*= $100*k*), (*V*)ehicle owner (therefore *V_i _*= 1). The estimate of distance for this profile at location (*u_i_*, *v_i_*) would be:

(14) $${\hat{d}}_{Y.H.V,i}=e^{{\hat{\beta }}_{0}+100{\hat{\beta }}_{1}+{\hat{\theta }}_{21}V_{i}+{\hat{\theta }}_{22}u_{i}V_{i}+{\hat{\theta }}_{23}v_{i}V_{i}}$$

In contrast, the estimate for a (*S*)enior (*S_i _*= 1), (*L*)ow income ($30*k*), (*N*)on-(*V*)ehicle owning (*V_i _*= 0) person would be:

(15) $${\hat{d}}_{S.L.NV,i}=e^{{\hat{\beta }}_{0}+30{\hat{\beta }}_{1}+{\hat{\theta }}_{11}S_{i}+{\hat{\theta }}_{12}u_{i}S_{i}+{\hat{\theta }}_{13}v_{i}S_{i}}$$

Naturally, the estimates for these personal profiles change when the equations are evaluated at a different location, say (*u_j_*, *v_j_*). Now a senior in the suburbs and a senior in the city centre may actually display differences in travel behavior. Flexible estimates of trip length obtained by means of the expansion method provide the basis for implementing relative accessibility analysis as outlined in the preceding section.

## Context and Data

**Figure 1:**
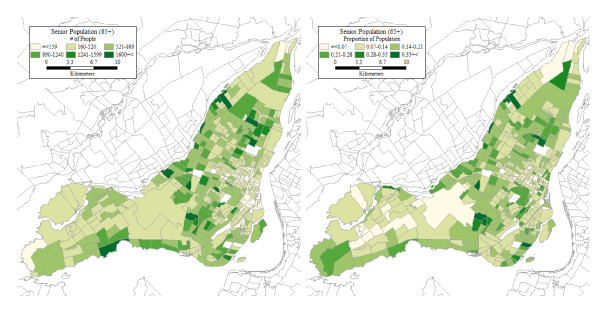
**Senior population in Montreal Island**.

The case study reported below is Montreal Island, part of the Greater Montreal Area (GMA) in Quebec, Canada. This is the second most populated urban area in Canada after Toronto, and the most populated in Quebec, where in fact it concentrates about half the population of the province. Between 2001 and 2006, the growth in population in the GMA was 5.3%, mainly due to immigration. During this period, growth followed sprawling development with more important gains in the inner and outer suburbs relative. In terms of the demographic composition, statistics of the *Institut de la Statistique du Québec *(ISQ) indicate that in 2007 14.4% of the population in Quebec was aged 65 years and older; this proportion exceeded 60% in some census subdivisions located in Montreal Island. Official projections estimate that the provincial population will increase from 7.65 million in 2006 to 8.11 million in 2031 (+9.6%), and a momentum towards aging will be maintained even after the population begins to decrease after 2031. According to ISQ projections, the proportion of seniors (65 years and older) in the province will rise to approximately 18% in 2016, 24% in 2026 and 31% in 2051. In the GMA specifically, recent research shows that the proportion of seniors already rose from 10.6% to 13.6% between 1987 and 2003, and that the spatial distribution of the senior population, while still more concentrated near the CBD, tended to disperse at a higher rate than the general population [56]. Figure 1 shows the distribution of the 2006 senior population in Montreal Island in absolute and proportional terms.

Three sources of data inform the analysis below. The first is Montreal's Household Travel Survey of 2003 (see http://www.cimtu.qc.ca/EnqOD/Index.asp). This is one of the largest cross-sectional origin-destination (OD) travel survey programs in the world, and has been conducted approximately every five years since 1970 in the GMA. In 2003 the survey was collected by means of Computer Aided Telephone Interviews with approximately 70,000 households or about 5% of all households residing in the survey area. The travel survey collects information on the individual travel behaviours of every person 5 years and older in the households interviewed, including number of trips, purpose, origins and destinations. In addition, the survey also records socio-economic and demographic information about the travellers. Place of residence and locations visited by an individual (home, trip-ends) are geocoded using structured databases on addresses, intersections, and trip generators. This allows for great flexibility in spatial analyses that can be conducted, either at the microdata level or at any level of aggregation, using any type of mapping delimitation. There are in total 122,420 records in the database corresponding to individuals who performed out-of-home activities during the day of the survey. The independent variable for the analysis is personal average trip length, defined as the total (straight line) distance travelled for all trips and purposes made during the day, divided by the number of trips. These calculations exclude the return-home trip. Straight line distance has the advantage of being simple to compute, and is highly correlated with network distance [29]. Explanatory variables are selected based on theoretical considerations and a survey of the previous literature on distance travelled. Further details about the survey and selection of variables can be found in [57], [38], and [36].

**Figure 2:**
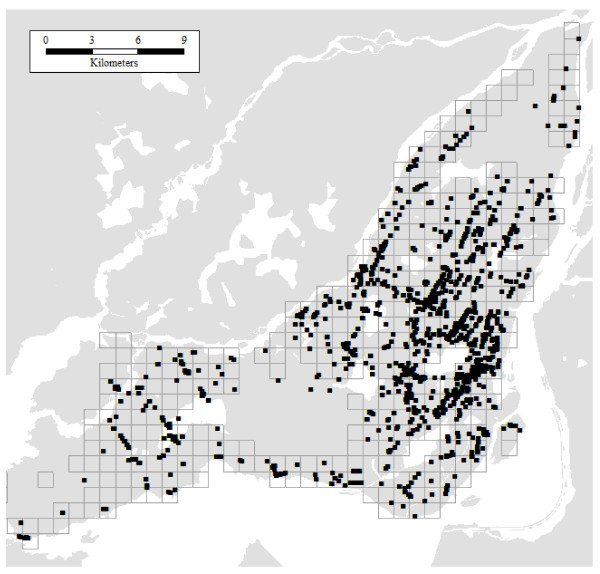
**Distribution of health care facilities in Montreal Island**.

The second source of information is a business point database. Business information is collected by infoCanada from a variety of sources, and verified annually for accuracy. Environics Analytics processes and packages the information, to create a georeferenced database with business profiles. This profile includes a Standard Industrial Classification code that can be used to identify various industries and business lines. Classification codes 8011 (Offices of Doctors of Medicine) and 8021 (Offices and Clinics of Dentists) were selected for extraction, but not laboratories or general medical, surgical, or specialty hospitals. Our selection of points is therefore more closely aligned with primary health care than with specialty care. There are 4,462 medical and dental offices in the Greater Montreal Area, and 2,593 in Montreal Island. As can be seen in Figure 2, these facilities tend to be concentrated, primarily in the central parts of Montreal Island. The final source of information was the Census of Canada, which was used to calculate population density as a proxy for urban form.

## Results and Discussion

### Estimates of Personal Average Trip Length

The results of estimating a model with expanded coefficients are shown in Table 1, with non-significant coefficients in italics. The value of the coefficient of determination for this model is 0.199, which is comparable to that reported for similar models in the literature [34], and in general indicates the amount of variability explained by the model. A full discussion of the geography of travel behaviour in Montreal can be found in Roorda et al. [38] and Morency et al. [36]. Average trip length in particular is conceptualized as a proxy for individual activity spaces [36]. Here we concentrate on some relevant highlights of the model. First, average trip length tends to decrease with decreasing income. This is noteworthy, because over 33% of seniors live in households with incomes below $20,000, and over 70% live in households with incomes below $40,000. Only about 3.3% of seniors live in households with incomes over one hundred thousand dollars.

**Table 1:** Regression model results.

**VARIABLE**	**Estimate**	**p-value**	**VARIABLE**	**Estimate**	**p-value**
	
CONSTANT	-2.1166	0.0000	**Urban form**		
	
**Age**			POPULATION DENSITY	-0.0218	0.0000
	
AGE < 20	-0.4165	0.0000	**Spatial expansion**		
AGE 20-35	0.0520	0.0000	DISTANCE TO CBD*^b^*	4.3285	0.0000
AGE 36-50	Reference		*Age 65+	-0.5318	0.0334
AGE 51-64	-0.0217	0.0144	*Single Parent	-1.3899	0.0024
AGE 65+	0.6027	0.0529	*Low Income	*-0.2927*	*0.1597*
			
**Income**			X^2^	-4.1085	0.0000
			
INC. REFUSE/DON'T KNOW	-0.1730	0.0000	*Age 65+	*0.6009*	*0.1901*
INCOME < 20 K	-0.9787	0.0080	*Single Parent	2.1477	0.0336
INCOME 20-40 K	-0.2513	0.0000	*Low Income	-2.7450	0.0000
INCOME 40-60 K	-0.1889	0.0000	X	5.6710	0.0000
INCOME 60-80 K	-0.1072	0.0000	*Age 65+	*-1.0814*	*0.1337*
INCOME 80-100 K	-0.0571	0.0001	*Single Parent	-3.7316	0.0105
INCOME > 100 K	Reference		*Low Income	3.3953	0.0003
			
**Household structure**			X*Y	-0.4131	0.0009
			
SINGLE	Reference		*Age 65+	*0.1262*	*0.3903*
COUPLE	*0.0105*	*0.1809*	*Single Parent	1.5921	0.0119
COUPLE W/CHILDREN	-0.1236	0.0000	*Low Income	*-0.4074*	*0.1887*
SINGLE PARENT	*0.3073*	*0.3242*	Y	4.8965	0.0000
OTHER	0.0429	0.0002	*Age 65+	-1.2045	0.0298
			
**Mobility tools**			*Single Parent	*-0.8996*	*0.2096*
			
DRIVER LICENSE	0.3061	0.0000	*Low Income	*0.0420*	*0.4768*
VEHICLE OWN	0.1699	0.0000	Y^2^	-5.4665	0.0000
*Age 65+	*-0.0036*	*0.4556*	*Age 65+	1.2649	0.0285
*Single Parent	*-0.0410*	*0.2030*	*Single Parent	1.7897	0.0611
*Low Income	*0.0159*	*0.2766*	*Low Income	*-0.3942*	*0.2974*
			
TRANSIT*^a^*	-0.0826	0.0001			
			
*Age 65+	-0.1679	0.0043	R^2^	0.199	
*Single Parent	*-0.1284*	*0.1488*	R^2^_adj_	0.198	
*Low Income	0.0925	0.0329	*s*^2^	1.208	
			
**Occupation**			*S*	1.099	
			
FULL TIME EMPLOYMENT	0.5701	0.0000	N	122420	
*Age 65+	-0.0953	0.0262			
*Single Parent	*0.0073*	*0.4278*			
*Low Income	-0.0602	0.0106			
PART TIME EMPLOYMENT	0.1674	0.0000			
*Age 65+	*0.0605*	*0.1878*			
*Single Parent	*0.0701*	*0.2369*			
*Low Income	0.1443	0.0006			
STUDENT	0.5323	0.0000			
FREE PARKING @ WORK	0.2271	0.0000			

Independent variable is log of average trip length

Notes:

*^a^*Major transit station within 500 m

*^b^*Central Business District

Secondly, vehicle ownership tends to increase average trip length for seniors and non-seniors alike. However, the vehicle ownership rate for households with seniors, at 74%, stands considerably below a rate of 89% for households without seniors. Lastly, in terms of household structure, the only significant results are the negative coefficient for couples with children and the positive coefficient for other types of multi-person households. The vast majority of seniors tend to live singly (31.3%) or as childless couples (51.6%), two types of household structure that are not significantly different from each other in terms of personal average trip length.

The positive and relatively large coefficient for age greater than 65 should be read with caution. This is the fixed component of a spatially expanded coefficient, and therefore must be assessed from the perspective of the net effect of the interaction between age and location. The net effect is more clearly appreciated by mapping the estimates of average trip length. In order to obtain estimates of distance travelled, we define four different profiles of interest, that we term REF (for the reference, i.e., non-senior group) and 65+ (for seniors). In addition, we examine the effect of vehicle ownership on average trip distance. The parameters used to define these four profiles appear in Table 2.

**Table 2:** Personal profiles for estimating average trip length

	PERSONAL PROFILES			
**VARIABLE**	**REF**	**REF&VEH**	**65+**	**65+&VEH**

**Age**				

AGE 36-50	✓	✓		
AGE 65+			✓	✓

**Income**				

INCOME 20-40 K			✓	✓
INCOME 40-60 K	✓	✓		

**Household structure**				

COUPLE	✓	✓	✓	✓

**Mobility tools**				

DRIVER LICENSE		✓		✓
VEHICLE OWN		✓		✓

**Occupation**				

FULL TIME EMPLOYMENT	✓	✓		

**Urban form**				

POPULATION DENSITY	✓	✓	✓	✓

**Spatial expansion**				

DISTANCE TO CBD	✓	✓	✓	✓
*Age 65+			✓	✓
X^2^	✓	✓	✓	✓
*Age 65+			✓	✓
X	✓	✓	✓	✓
*Age 65+			✓	✓
X*Y	✓	✓	✓	✓
*Age 65+			✓	✓
Y	✓	✓	✓	✓
*Age 65+			✓	✓
Y^2^	✓	✓	✓	✓
*Age 65+			✓	✓

Personal average trip length can be estimated at any point within the region covered by the model. For visualization purposes we choose to use a regular grid with square cells of 1 km^2 ^covering the populated areas of Montreal Island according to the Household Travel Survey. The coordinates of the grid cell centroids are used to calculate our estimates of distance travelled, in conjunction with the coefficients in Table 1 and the designated personal profiles in Table 2. Finer grids provide more detailed geographically estimates, but do not change the general picture. For our purposes, this resolution is adequate.

**Figure 3:**
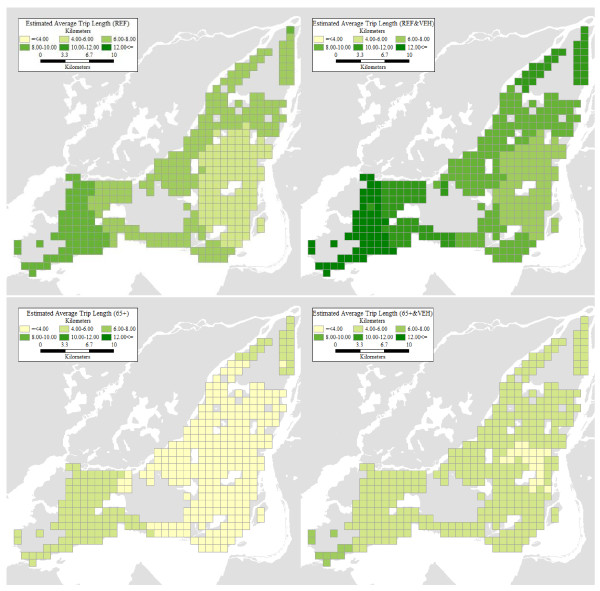
**Spatial estimates of average trip length for four personal profiles**.

Figure 3 shows the results of calculating our estimates of average trip length. The geographical pattern is similar for the four profiles, with shorter trip lengths in the central parts of Montreal Island, and increasingly lengthier trips towards the suburban parts of the region. In concordance with the literature on aging and mobility [see [39]], seniors tend to have significantly lower levels of mobility. However, as the figures clearly illustrate, this is particularly true of seniors in suburban settings, and seniors without vehicles. Different mobility levels are expected to be reflected in the levels of personal accessibility at various locations.

### Accessibility Levels and Relative Accessibility

**Figure 4:**
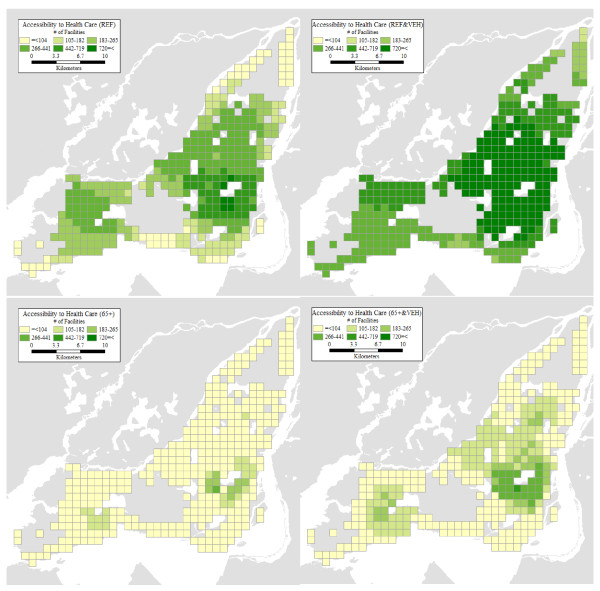
**Accessibility levels for four personal profiles**.

In this section, we present the results of our accessibility analyses. For each grid cell centroid and personal profile, we use the corresponding estimate of average trip distance as the flexible bandwidth to count the number of accessible opportunities as per Equation. The levels of accessibility are displayed in Figure 4. It can be seen there that the geographical patterns are broadly similar for the four personal profiles, with higher accessibility levels predominant in the centre of Montreal Island, and to some extent also in the southwest parts of the island. The particulars, however, could not be more different. Seniors tend to have quite low levels of accessibility especially in the suburbs, despite having relatively higher levels of mobility there (see Figure 3). Even with a vehicle, their levels of accessibility remain woefully below those corresponding to the reference personal profile. This is unfortunate because, as shown in Figure 1, many of the places with large concentrations of seniors in absolute and proportional terms are also those where accessibility tends to be lower for seniors.

**Figure 5:**
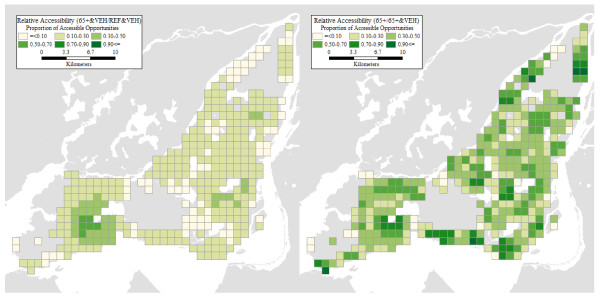
**Relative accessibility: a) 65+&VEH/REF&VEH; b) 65+/65+&VEH**.

Two maps in Figure 5 illustrate just how large are the differences in accessibility between population profiles. The first map in the figure is the relative accessibility deprivation indicator of seniors with vehicle, relative to the reference profile with vehicle. Recall that this indicator is a proportion of the number of opportunities accessible to an individual of the designated profile (e.g. senior with a vehicle), relative to the opportunities available to a comparison profile (e.g. reference with vehicle). As seen in the figure, nowhere are seniors close to accessibility parity with the reference. At best, a senior has access to 70% of the opportunities available to the reference profile, but this is in a circumscribed region in the southwest part of the Island, where accessibility is only moderately high to begin with. The second map illustrates the accessibility effects for seniors of vehicle ownership, and shows the relative accessibility deprivation indicator for seniors without vehicle relative to those with vehicles. The disparities here are less glaring but no less important, especially because values closer to parity are observed for the most part in areas where the base level of accessibility is low to begin with. In other words, in these areas even a vehicle is not sufficient to greatly improve the accessibility of seniors to health care facilities.

### Accessibility Levels Using a Fixed Bandwidth

**Figure 6:**
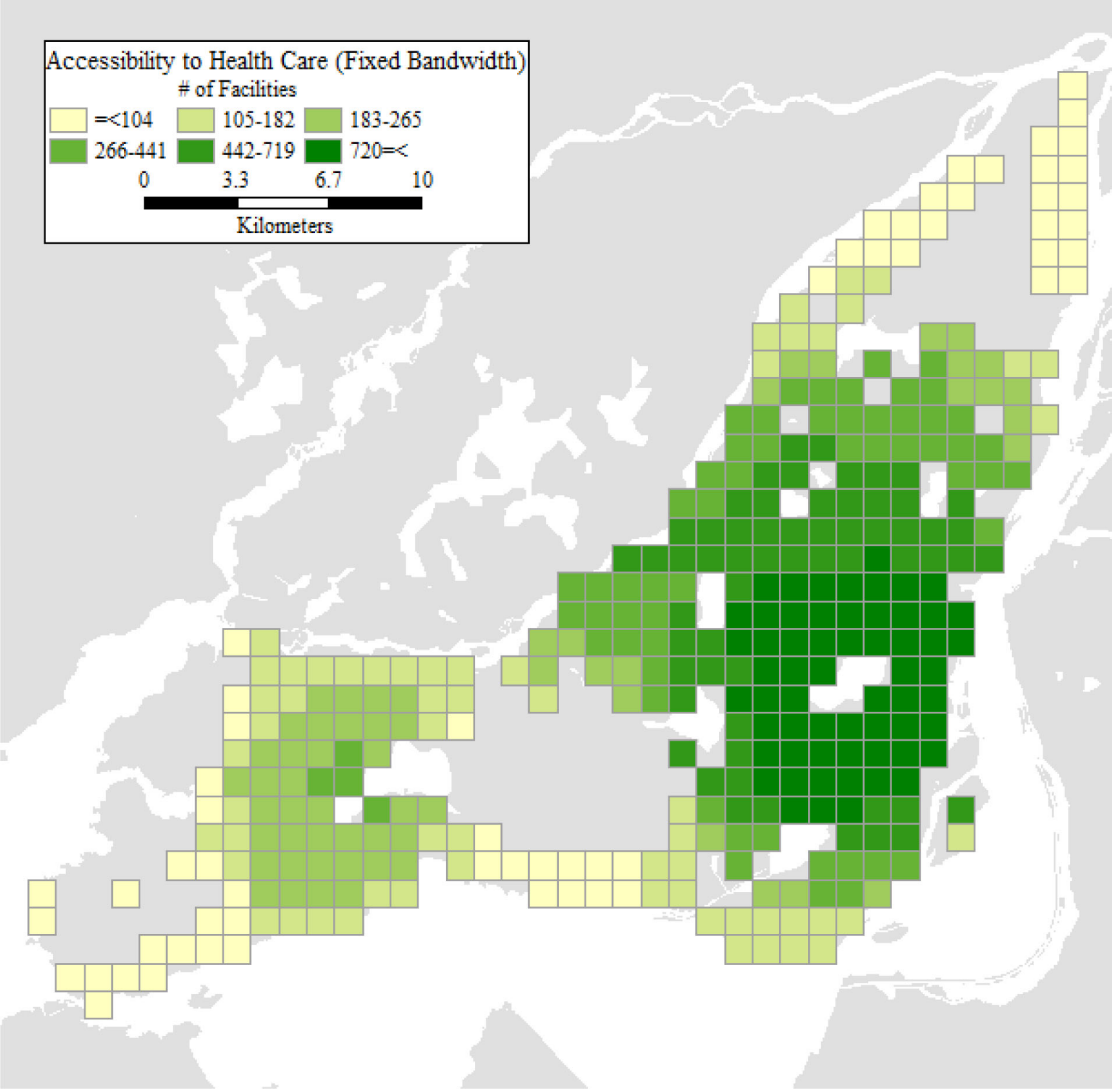
**Accessibility levels using fixed bandwidth (4.8 km)**.

Besides issues of validation, and a lack of consensus regarding an appropriate value for calculating accessibility using a fixed bandwidth, relative accessibility analysis offers the advantage of providing more nuanced and detailed results. As an example, in Figure 6 we show the levels of accessibility that are obtained by adopting a fixed bandwidth of 4.8 km. This value was used by Guagliardo et al. [48], and is relatively conservative, considering that some standards call for bandwidths of over 20 km. However, our main concern is not the use of a specific value for the bandwidth, but the lack of geographical, socio-economic, and demographic variations associated with the use of fixed bandwidths. As seen in Figure 6, a fixed bandwidth is in effect a one-size-fits-all approach to accessibility analysis. While the 4.8 km bandwidth coincidentally approximates the levels of accessibility of the profile REF (without a vehicle) it still tends to slightly overestimate the accessibility for this profile in the central city and underestimate it in the suburbs. The differences are more dramatic for other profiles. Accessibility levels calculated with a 4.8 km bandwidth tend to be overly optimistic for the case suburban seniors. Regardless of vehicle ownership status, suburban seniors tend to have considerably lower levels of accessibility than suggested by a bandwidth of 4.8 km, once their mobility patterns and evidence of the "transportation burdens" they face are taken into account. The levels of accessibility obtained by the means of a fixed bandwidth analysis suggest levels of accessibility that would in fact be difficult to achieve by typical seniors, given their actual mobility patterns. Contrariwise, a bandwidth of 4.8 km provides an unduly pessimistic view of accessibility to health care for vehicle owning individuals of the reference profile in the suburbs.

## Discussion and Conclusions

In this paper we demonstrate the use of relative accessibility deprivation indicators to investigate access to health care facilities from the perspective of seniors and non-seniors in Montreal Island. Unlike conventional approaches that assume a fixed bandwidth for all accessibility calculations, use of travel behaviour information provides more refined estimates of accessibility that take into account variations in the burden of transportation, as experienced by a variety of individuals. The use of a spatial modelling approach provides statistically valid estimates for the bandwidth parameter, as opposed to assumed values that may or may not bear relationship to the actual patterns of mobility of the public. Furthermore, the use of flexible bandwidths also allows us to conduct inter-personal and geographical comparisons. Indeed, the results of our analyses show that there are important (and statistically significant) variations in the levels of mobility and accessibility of seniors in various locations, as well as in relation to a designated reference group. These differences, which would be poorly approximated by the use of a fixed bandwidth, suggest that accessibility to health care facilities in Montreal Island tends to be lower precisely in many of the places where seniors tend to be more numerous. Some regions are identified where vehicle ownership is not sufficient to increase the level of accessibility of seniors.

The indicator of travel behaviour selected for this study was average trip length. This is the typical distance that a person covers as part of one displacement in a day. We prefer the use of this indicator, calculated using all trip purposes, to obtain an all-purpose summary measure of the spaces of daily life. This is not to say that people will not occasionally travel longer distances. However, thinking about accessibility, this means that reaching further opportunities would already exceed the distance that an individual would typically travel for any one trip. While different criteria could be adopted, we would submit that the flexibility of using estimates of average trip length already represents an important step forward relative to current practice.

One important distinction that should be evident to readers familiar with the accessibility literature, but that nonetheless bears remarking again, is that between accessibility (the potential for reaching destinations) and access (a specific realization of that potential). Even at the lowest levels of accessibility, for instance in the case of seniors without vehicles, some health care facilities are available within the reach of a typical trip. Even considering that seniors are less likely to perform at least one out-of-home activity on a given day [38], this means that health care facilities are not completely absent. In this respect, it is important to keep in mind the following points: 1) relative accessibility analysis shows that seniors have potential access to fewer opportunities than more mainstream segments of the population, which places them at risk of social exclusion [58]; in addition 2) when accessibility is low, choice may be more restricted; and finally 3) suburban travellers tend to make longer trips in areas where accessibility is low and the density of opportunities is also generally lower, which can make trip chaining more challenging. As with any analysis involving potential accessibility, a careful assessment of the implications must avoid unwarranted conclusions about actual access, or the levels at which accessibility becomes inadequate. We suggest that the results of our analysis could be used as a proxy for access in statistical investigations of health outcomes. Another possibility is to use the results to inform the selection of sites for more in-depth studies, for instance to target purposive data collection efforts to assess the adequacy of health care utilization and the impact on outcomes.

## Competing interests

The authors declare that they have no competing interests.

## Authors' contributions

All authors contributed equally to the conceptual framework for this study. Statistical analysis of distance travelled was performed by AP and CM. Accessibility analysis was conducted by AP. Background research was performed by RGM and SF. AP produced a first draft of the manuscript. All authors read and approved the final manuscript.

## References

[B1] Peters DH Garg A Bloom G Walker DG Brieger WR Rahman MH Poverty and access to health care in developing countries Annals of the New York Academy of Sciences 2008 1136 161 171 10.1196/annals.1425.011 17954679

[B2] McCormick MC Weinick RM Elixhauser A Stagnitti MN Thompson J Simpson L Annual report on access to and utilization of health care for children and youth in the United States-2000 Ambulatory Pediatrics 2001 1 3 15 10.1367/1539-4409(2001)001<0003:AROATA>2.0.CO;2 11888366

[B3] Reed MC Tu HT Triple jeopardy: low income, cronically ill and insured in America Issue Brief (Center for Studying Health System Change 2002 49 1 4 11865909

[B4] Broyles RW Narine L Brandt EN The temporarily and chronically uninsured: Does their use of primary care differ? Journal of Health Care for the Poor and Underserved 2002 13 95 111 11836917 10.1353/hpu.2010.0159

[B5] Cunningham PJ Declining employer-sponsored coverage: The role of public programs and implications for access to care Medical Care Research and Review 2002 59 79 98 11877880 10.1177/107755870205900104

[B6] Guyer J Broaddus M Dude A Millions of mothers lack health insurance coverage in the United States - Most uninsured mothers lack access both to employer-based coverage and to publicly subsidized health insurance International Journal of Health Services 2002 32 89 106 10.2190/D6T9-8P8Y-TT8L-RPP6 11913859

[B7] Porell FW Miltiades HB Access to care and functional status change among aged Medicare beneficiaries Journals of Gerontology Series B-Psychological Sciences and Social Sciences 2001 56 S69 S83 10.1093/geronb/56.2.s69 11245367

[B8] Hoffman C Paradise J Health insurance and access to health care in the United States Annals of the New York Academy of Sciences 2008 1136 149 160 10.1196/annals.1425.007 17954671

[B9] Fitzpatrick AL Powe NR Cooper LS Ives DG Robbins JA Barriers to health care access among the elderly and who perceives them American Journal of Public Health 2004 94 1788 1794 10.2105/AJPH.94.10.1788 15451751 PMC1448535

[B10] Schneider EC Zaslavsky AM Epstein AM Racial disparities in the quality of care for enrolees in Medicare managed care Journal of the American Medical Association 2002 287 1288 1294 10.1001/jama.287.10.1288 11886320

[B11] Gormick ME The association of race/socioeconomic status and use of Medicare: A little-known failure in access to care Annals of the New York Academy of Sciences 1999 896 497 500 10.1111/j.1749-6632.1999.tb08180.x 10681961

[B12] Janes JR Blackman DK Bolen JC Surveillance for use of preventive health-care services by older adults, 1995-1997 MMWR CDC Surveillance Summary 1999 48 51 88 10634271

[B13] Hsia J Kemper E Sofaer S Is insurance a more important determinant of health care access than perceived health? Evidence from the Women's Health Initiative Journal of Women's Health and Gender-Based Medicine 2009 9 881 889 10.1089/152460900750020919 11074954

[B14] Escarce JJ Epstein KR Colby DC Schwartz JS Racial differences in elderly's use of medical procedures and diagnostic tests American Journal of Public Health 1993 83 948 954 10.2105/AJPH.83.7.948 8328615 PMC1694780

[B15] Bostock L Pathways of disadvantage? Walking as a mode of transport among low-income mothers Health & Social Care in the Community 2001 9 11 18 10.1046/j.1365-2524.2001.00275.x 11560717

[B16] Litwin H Landau R Social network type and social support among the old-old Journal of Aging Studies 2000 14 213 228 10.1016/S0890-4065(00)80012-2

[B17] Phillips J Bernard M Phillipson C Ogg J Social support in later life: A study of three areas British Journal of Social Work 2000 30 837 853 10.1093/bjsw/30.6.837

[B18] Rittner B Kirk AB Health-Care and Public Transportation Use by Poor and Frail Elderly People Social Work 1995 40 365 373 7761923

[B19] Lai DWL Chau SBY Predictors of Health Service Barriers for Older Chinese Immigrants in Canada Health and Social Work 2007 32 57 65 17432742 10.1093/hsw/32.1.57

[B20] Exworthy M Peckham S Access, choice and travel: Implications for health policy Social Policy & Administration 2006 40 267 287

[B21] Dolan TA Atchinson K Huynh TN Access to Dental Care Among Older Adults in the United States Journal of Dental Education 2005 69 961 974 16141082

[B22] Wang FH Luo W Assessing spatial and nonspatial factors for healthcare access: towards an integrated approach to defining health professional shortage areas Health & Place 2005 11 131 146 10.1016/j.healthplace.2004.02.003 15629681

[B23] Guagliardo MF Spatial accessibility of primary care: concepts, methods and challenges International Journal of Health Geographics 2004 3 1 13 10.1186/1476-072X-3-3 14987337 PMC394340

[B24] Goodman DC Mick SS Bott D Stukel T Chang CH Marth N Primary Care Service Areas: A new tool for the evaluation of primary care services Health Services Research 2003 38 287 309 10.1111/1475-6773.00116 12650392 PMC1360885

[B25] Wang FH Luo W Assessing spatial and nonspatial factors for healthcare access: towards an integrated approach to defining health professional shortage areas Health & Place 2005 11 131 146 10.1016/j.healthplace.2004.02.003 15629681

[B26] Luo W Qi Y An enhanced two-step floating catchment area (E2SFCA) method for measuring spatial accessibility to primary care physicians Health & Place 2009 15 1100 1107 10.1016/j.healthplace.2009.06.002 19576837

[B27] Ray N Ebener S AccessMod 3.0: computing geographic coverage and accessibility to health care services using anisotropic movement of patients International Journal of Health Geographics 2008 7 1 17 10.1186/1476-072X-7-63 19087277 PMC2651127

[B28] Schuurman N Berube M Crooks VA Measuring potential spatial access to primary health care physicians using a modified gravity model Canadian Geographer-Geographe Canadien 2010 54 29 45 10.1111/j.1541-0064.2009.00301.x

[B29] Apparicio P Abdelmajid M Riva M Shearmur R Comparing alternative approaches to measuring the geographical accessibility of urban health services: Distance types and aggregation-error issues International Journal of Health Geographics 2008 7 10.1186/1476-072X-7-7 PMC2265683 18282284

[B30] Gu W Wang X McGregor SE Optimization of preventive health care facility locations International Journal of Health Geographics 2010 9 1 16 10.1186/1476-072X-9-17 20298608 PMC3161374

[B31] Pagano E Di Cuonzo D Bona C Baldi I Gabriele P Ricardi U Accessibility as a major determinant of radiotherapy underutilization: A population based study Health Policy 2007 80 483 491 10.1016/j.healthpol.2006.05.006 16781002

[B32] Paez A Scott D Potoglou D Kanaroglou P Newbold KB Elderly mobility: Demographic and spatial analysis of trip making in the Hamilton CMA, Canada Urban Studies 2007 44 123 146 10.1080/00420980601023885

[B33] Schwanen T Dijst M Dieleman FM Leisure trips of senior citizens: Determinants of modal choice Tijdschrift Voor Economische en Sociale Geografie 2001 92 347 360 10.1111/1467-9663.00161

[B34] Schmocker JD Quddus MA Noland RB Bell MGH Estimating trip generation of elderly and disabled people - Analysis of London data Transportation Research Record 2005 9 18 10.3141/1924-02

[B35] Mercado RG Páez A Determinants of distance traveled with a focus on the elderly: A multilevel analysis in the Hamilton CMA, Canada Journal of Transport Geography 2009 17 65 76 10.1016/j.jtrangeo.2008.04.012

[B36] Morency C Páez A Roorda MJ Mercado RG Farber S Distance traveled in three Canadian cities: Spatial analysis from the perspective of vulnerable population segments Journal of Transport Geography 2010 in press

[B37] Pettersson P Schmöcker JD Active ageing in developing countries? - trip generation and tour complexity of older people in Metro Manila Journal of Transport Geography 2010 18 613 623 10.1016/j.jtrangeo.2010.03.015

[B38] Roorda MJ Páez A Morency C Mercado RG Farber S Trip generation of vulnerable populations in three Canadian cities: A spatial ordered probit approach Transportation 2010 37 525 548 10.1007/s11116-010-9263-3

[B39] Schwanen T Páez A The mobility of older people - an introduction Journal of Transport Geography 2010 18 591 595 10.1016/j.jtrangeo.2010.06.001

[B40] Harrison A Ragland DR Consequences of driving reduction or cessation for older adults Transportation Research Record 2003 96 104 10.3141/1843-12

[B41] Davey JA Older people and transport: coping without a car Ageing & Society 2007 27 49 65

[B42] Alsnih R Hensher DA The mobility and accessibility expectations of seniors in an aging population Transportation Research Part A-Policy and Practice 2003 37 903 916 10.1016/S0965-8564(03)00073-9

[B43] Páez A Mercado RG Farber S Morency C Roorda M Relative Accessibility Deprivation Indicators for Urban Settings: Definitions and Application to Food Deserts in Montreal Urban Studies 2010 47 1415 1438 10.1177/0042098009353626

[B44] Handy S Niemeier D Measuring Accessibility: An Exploration of Issues and Alternatives Environment and Planning A 1997 29 1175 1194 10.1068/a291175

[B45] Kwan MP Space-time and integral measures of individual accessibility: A comparative analysis using a point-based framework Geographical Analysis 1998 30 191 216 10.1111/j.1538-4632.1998.tb00396.x

[B46] Geurs KT van Wee B Accessibility evaluation of land-use and transport strategies: review and research directions Journal of Transport Geography 2004 12 127 140 10.1016/j.jtrangeo.2003.10.005

[B47] O'Kelly ME Horner MW Aggregate accessibility to population at the county level: U.S. 1940-2000 Journal of Geographical Systems 2003 5 5 23 10.1007/s101090300101

[B48] Guagliardo MF Ronzio CR Cheung I Chacko E Joseph JG Physician accessibility: an urban case study of pediatric providers Health & Place 2004 10 273 283 10.1016/j.healthplace.2003.01.001 15177201

[B49] Shannon GW Bashshur RL Spurlock CW Search for Medical-Care - Exploration of Urban Black Behavior International Journal of Health Services 1978 8 519 530 681049 10.2190/0F28-23GM-42K0-PQDN

[B50] Luo W Wang FH Measures of spatial accessibility to health care in a GIS environment: synthesis and a case study in the Chicago region Environment and Planning B-Planning & Design 2003 30 865 884 10.1068/b29120 PMC8238135 34188345

[B51] Lee RC Current approaches to shortage area designation Journal of Rural Health 1991 7 437 450 10116034

[B52] Wang FH McLafferty S Escamilla V Luo L Late-stage breast cancer diagnosis and health care access in illinois Professional Geographer 2008 60 54 69 10.1080/00330120701724087 18458760 PMC2367325

[B53] Haynes R Lovett A Sunnenberg G Potential accessibility, travel time, and consumer choice: geographical variations in general medical practice registrations in Eastern England Environment and Planning A 2003 35 1733 1750 10.1068/a35165

[B54] Schönfelder S Axhausen KW Activity spaces: measures of social exclusion? Transport Policy 2003 10 273 286 10.1016/j.tranpol.2003.07.002

[B55] Casetti E Generating Models by the Expansion Method: Applications to Geographic Research Geographical Analysis 1972 28 281 298

[B56] Morency C Chapleau R Age and its relation with home location, household structure and travel behaviors: 15 years of observation87th Annual Meeting of the Transportation Research Board; 13 January, 2008; Washington, D.C 2008

[B57] Páez A Mercado RG Farber S Morency C Roorda M Mobility and Social Exclusion in Canadian Communities: An Empirical Investigation of Opportunity Access and Deprivation Report to Policy Research Directorate, Strategic Policy and Research, Human Resources and Social Development Canada 2009 http://www.science.mcmaster.ca/geo/faculty/paez/publications.html#reports

[B58] Social Exclusion Unit Making the Connections: Final Report on Transportation and Social Exclusion 2003 London: HMSO

